# Editorial: Artificial intelligence and social equities: navigating the intersectionalities in a digital age

**DOI:** 10.3389/fsoc.2026.1951102

**Published:** 2026-08-26

**Authors:** Daisuke Akiba, Julie Albright

**Affiliations:** 1 Queens College and the Graduate Center, The City University of New York, New York City, NY, United States; 2 University of Southern California, Los Angeles, CA, United States

**Keywords:** AI, AI and human rights, algorithmic bias and discrimination, cultural computing, digital equity, digital inclusivity, ethical AI governance, intersectionality in technology

As artificial intelligence (AI) transitions from theoretical computer science into the heart of modern social infrastructure, its *societal footprint* has expanded exponentially. Technologies such as large language models, computer vision, and predictive analytics hold immense promise for addressing systemic global challenges. However, if not approached with care, they can simultaneously risk encoding, automating, or even amplifying sociohistorical inequities.

This Research Topic, co-edited across the behavioral, social, and computer sciences, was established to explore the multifaceted intersections of AI with race, ethnicity, and interconnected identity dimensions. The current collaborative effort interrogates both the risks of algorithmic systems and their transformative potential as tools for promoting equity, pluralism, and intergroup understanding. The eight contributions published in this Research Topic offer a timely, globally diverse exploration of these dynamics, bridging empirical research, theoretical frameworks, and prospective analyses.

## Algorithmic injustice and epistemic inequities

A primary focus of this Research Topic centers on how automated systems reinforce structural disadvantage across marginalized populations. Fields et al. address algorithmic bias in mental health by proposing an ethical governance framework for anti-racist AI in healthcare. By explicitly incorporating racism-related stress into psychiatric algorithms for Black Americans, the authors demonstrate how clinical models can move beyond harmful color-blindness toward culturally responsive design.

Expanding the discussion to digital media, Putri offers an intersectional mini-review examining how AI-generated visual disinformation disproportionately impacts marginalized groups. This work highlights how algorithmic vulnerabilities exploit pre-existing social fault lines, deepening digital divides. On a global scale, Olaniyan et al. investigate how generative AI influences epistemic (in)justice (i.e., the fairness with which different groups' knowledge and testimony are credited) among higher education students across the Global North and Global South. Their findings reveal stark geopolitical divides in how AI-mediated knowledge production is experienced.

## Identity, culture, and power dynamics

Algorithms do not operate in a vacuum; they interact dynamically with localized cultural contexts, regional economies, and societal structures. Yuehua and Jiayao quantify the digital cultural divide in China, evaluating how platform algorithms actively shape rural–urban identity politics. Their empirical work demonstrates that recommendation engines can heighten regional polarization by commodifying rural identities for urban consumption. In the domain of organizational communication, Sonni critically reviews AI-enhanced communication strategies within Southeast Asian startup ecosystems, showing how adopting westernized AI models without cultural translation can inadvertently erase regional communication nuances and marginalize local talent.

Looking beyond present-day systems, Singh and Sabu present a conceptual analysis mapping discrimination in “futuristic societies” through the lens of speculative fiction. By tracing the transition from human bias to posthuman bias, they challenge us to contemplate how emerging technologies might construct entirely new classes of systemic oppression if left unchecked.

## Frameworks for governance, accessibility, and human centricity

Addressing these systemic challenges requires robust theoretical and practical frameworks for design, verification, and ethical deployment. Waters contributes a theoretical framework for AI testing, evaluation, verification, and validation (TEVV; the audit processes by which a system's performance and safety are certified) focused on accessibility, arguing that true accessibility in digital health and public platforms cannot be an afterthought; it must be systematically audited across every stage of the AI development lifecycle. Finally, addressing the fundamental tension between algorithmic efficiency and human empathy, Schenk explores the permissibility of AI through the concepts of mechanical objectivity (i.e., rule-bound, standardized decision-making that deliberately excludes human judgment) and empathic understanding, challenging the uncritical adoption of automated decision-making in contexts where efficiency must not replace human nuance and moral judgment.

## Synthesis and future directions

Taken together, these eight articles illustrate that AI is neither inherently empowering nor inherently oppressive. Rather, its impact is mediated by the socioeconomic, cultural, and political conditions under which it is developed and deployed. Structural risks (e.g., algorithmic bias, epistemic injustice, digital divides, etc.) and equity-driven approaches (e.g., culturally informed models, accessible TEVV frameworks, intersectional design, etc.), thus, jointly shape AI's consequences for racial and ethnic equity.

Three cross-cutting insights emerge here. First, algorithmic harm is rarely a purely technical failure; rather, it is a structural phenomenon rooted in whose data, language, and lived experiences are treated as the default. Although this insight has been well-established in critical scholarship on algorithmic bias, the distinctive contribution of the present Research Topic lies in tracing how it operates within specific high-stakes domains, such as psychiatric care for Black Americans (Fields et al.) and knowledge production across the Global North and South (Olaniyan et al.). Second, equity cannot be retrofitted: culturally responsive design, accessibility auditing, and intersectional analysis must be embedded throughout the AI lifecycle (i.e., from problem formulation and data curation through validation and post-deployment monitoring) rather than appended after harm has occurred (Sonni; Waters). Third, context is constitutive: identical technologies yield divergent outcomes across regions, communities, and political economies, cautioning against the wholesale export of one-size-fits-all models across cultural boundaries (Yuehua and Jiayao; Putri; Schenk). These convergent themes, nevertheless, should not obscure the Research Topic's methodological heterogeneity: some contributions ground their claims in empirical data while others advance conceptual frameworks whose propositions await empirical validation. Additionally, results drawn from single countries or contexts should be generalized with caution.

Equally meaningful is what remains underexplored. This Research Topic invited work on AI in asylum processing and migration governance, on inclusive education, and on communities where identities or help-seeking behaviors are stigmatized or criminalized; yet, these domains remain underrepresented in the present Research Topic and, thus, constitute urgent priorities for future scholarship, as do the intersections of race and ethnicity with religion, disability, and other identities, particularly in non-Western contexts.

Building on the foundations laid here, we call on future research to: develop community-led ethical frameworks that center marginalized voices from the design phase onward; expand intersectional methodologies (i.e., quantitative, qualitative, and mixed) that capture the compounding effects of race, ethnicity, socioeconomic status, gender, and disability; implement enforceable auditing and TEVV standards for AI in high-stakes domains including healthcare, education, border control, and public policy; and pursue longitudinal, cross-national comparative studies that track equity impacts as technologies and regulatory landscapes co-evolve.

We trust this Research Topic serves as both a comprehensive reference for scholars and a catalyst for actionable, equity-driven AI governance. Clearly, the stakes are concrete: as algorithms increasingly mediate access to care, education, and opportunity, design choices will translate directly into the racial and ethnic disparities these contributions seek to redress.

